# Insights into the *Vitis* complex in the Danube floodplain (Austria)

**DOI:** 10.1002/ece3.3187

**Published:** 2017-08-28

**Authors:** Claire Arnold, Olivier Bachmann, Annik Schnitzler

**Affiliations:** ^1^ Unicentre, University of Lausanne Lausanne Switzerland; ^2^ Laboratory of Evolutionary Botany University of Neuchâtel Neuchâtel Switzerland; ^3^ Laboratory of Plant Ecology University of Basel Basel Switzerland; ^4^ Laboratoire Interdisciplinaire des Environnements Continentaux LIEC ‐ UMR 7360 CNRS University of Lorraine Metz France

**Keywords:** genetic diversity, habitat fragmentation, invasion biology, river dynamics, *Vitis* complex

## Abstract

European grapevine populations quickly disappeared from most of their range, massively killed by the spread of North American grapevine pests and diseases. Nowadays taxonomic pollution represents a new threat. A large *Vitis* complex involves escaped cultivars, rootstocks, and wild grapevines. The study aimed to provide insight into the *Vitis* complex in the Danube region through field and genetic analyses. Among the five other major rivers in Europe which still host wild grapevine populations, the Danube floodplain is the only one benefiting from an extensive protected forest area (93 km²) and an relatively active dynamic flood pulse. The Donau‐Auen National Park also regroups the largest wild grapevine population in Europe. Ninety‐two percent of the individuals collected in the park were true wild grapevines, and 8% were hybrids and introgressed individuals of rootstocks, wild grapevines, and cultivars. These three groups are interfertile acting either as pollen donor or receiver. Hybrids were established within and outside the dykes, mostly in anthropized forest edges. The best‐developed individuals imply rootstock genes. They establish in the most erosive parts of the floodplain. 42% of the true wild grapevines lived at the edges of forest/meadow, 33.3% at the edges forest/channels, and 23.9% in forest gaps. DBH (Diameter Breast Height) varied significantly with the occurrence of flooding. Clones were found in both true wild and hybrids/introgressed grapevines. The process of cloning seemed to be prevented in places where flooding dynamics is reduced. The current global distribution of true wild grapevines shows a strong tendency toward clustering, in sites where forestry practices were the most extensive. However, the reduced flooding activity is a danger for long‐term sustainability of the natural wild grapevine population.

## 1 INTRODUCTION

**Figure 1:**
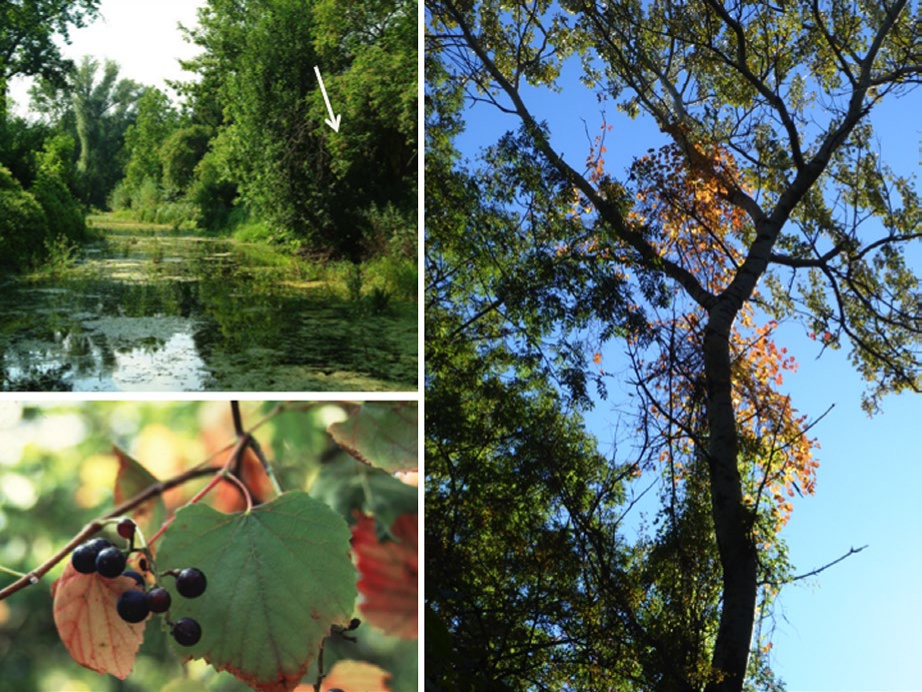
Typical habitat of wild grapevine in the Donau‐Auen National Park (upper left). Wild grapevine in the canopy in autumn (right). Grape berries of a female wild grapevine (lower left) Photographs by Claire Arnold and Olivier Bachmann

The Eurasian wild grapevine (*Vitis vinifera* ssp. *sylvestris* (Gmelin) Hegi) is currently distributed in a few alluvial (Figure 1) and colluvial forests around the Mediterranean basin between the 38th and 49th northern parallel, from sea level up to an altitude of 1,600 m (Arnold, 2002; Vassilczenko, 1970). These areas are refugia where grapevine pest (the homoptera *Daktulosphaira vitifoliae* Ficht traditionally called phylloxera) and fungi diseases (oïdium; mildew) have a restricted spread. These pest and diseases were imported with the American *Vitis* species at the end of the 19th century.

Phylloxera is particularly harmful for grapevine. It has been the major factor in determining the rate of decline in vineyards and wild populations worldwide since the middle of the 19th century (Arnold, 2002). Grapevines survived in wet, temporarily anoxic soils of alluvial areas where this homoptera could not live ( et al., 2004a; Ocete et al., 2006). River management led to the elimination of flood events, and a sinking of ground water levels. This induced among others a severe drying out of the environment. Phylloxera could then enter floodplains and killed massively grapevine populations (Arnold, 2002). For example in the Rhine upper valley, the 200 individuals recorded at the beginning of the 20th century (Issler, 1938; Kirchheimer, 1946; Schutz, 1946) had nearly disappeared a few decades later (Arnold, Schnitzler, Douard, Peter, & Gillet, 2005; Schumann, 1974). In Austria, Jacquin (1762) described forests covered with veils of grapevines. In 1955, Kirchheimer gave an update of the presence of wild grapevines in Lower Austria and considered this species in decline because of the destruction of its habitats. In 1972, wild grapevines just remained on the left riverside of the Danube and only downstream Vienna (Ehrendorfer & Niklfeld, 1972).

Recent studies have shown that wild grapevines survived as small populations in remote mountain sites, screes, floodplain forests of large rivers, their deltas, and their tributaries (Danube, Rhine, Rhône, Seine, Guadalquivir, Pô), in no‐man's‐lands between countries, and on islands (Corsica, Sardinia) (Anzani, Failla, Scienza, & Campostrini, 1990; Arnold, 2002; Arnold, Gillet, & Gobat, 1998; Arrigo & Arnold, 2007; Arroyo‐Garcia et al., 2006; Lacombe et al., 2003; Ocete et al., 2004a,2004b; Terpo, 1976). In light of the ongoing threats, *V. vinifera* ssp. *sylvestris* has thus been considered as an “endangered species” since the 1980s.

Another threat has to be taken into account: taxonomic pollution through gene flows between wild grapevines and the *Vitis* taxa that escape from vineyards. The taxa may be either European cultivars (*V. vinifera* ssp. *vinifera)*, interspecific cultivars (PIWI (pilzwiderstandsfähig) (a total of 6,154 cultivars have been created in the world, OIV, 2013) or artificial polyhybrids of *Vitis* species (*Vitis aestivalis, V. berlandieri, V. cinerea, V. labrusca, V. riparia, V. rupestris)* that are used as rootstocks for grafting onto cultivars. Specific rootstocks are used in each viticultural region according to the local abiotic conditions, such as calcium, salt, lime, or clay content of soils. When they escape from vineyards, they rapidly invade unoccupied lands or anthropized landscapes (roadsides, channels, railroad tracks) via sexual and vegetative reproduction. These rootstocks have good rooting capacity and large leaves, and they produce a large amount of fruits. Anthropogenic populations can therefore rapidly cover large surfaces. They are also resistant but are vectors of pathogens and diseases. As all the *Vitis* species in the world seem to be interfertile and show a remarkable ability to hybridize with sister species (Arroyo‐Garcia et al., 2006; Levadoux, 1956; Tröndel et al., 2010), grapevines found in the wild are considered to be a mixture of wild forms, naturalized cultivars and rootstocks, and hybrids derived from spontaneous hybridizations and introgressions among these species and forms (Arrigo & Arnold, 2007; Bodor et al., 2010; Lacombe et al., 2003; Laguna, 2003; Laguna 2004; Levadoux, 1956; Lowe & Walker, 2006; Ocete et al., 2012; This, Lacombe, & Thomas, 2006; Warwick & Stewart, 2005; Zecca et al., 2009).

Two aspects of the *Vitis* complex dynamics have not yet been investigated in‐depth: (1) the contribution of parents (i.e., orientation of crossings and parentage pedigree) and (2) the role of habitat characteristics in the propagation and establishment of progenies in nature. Indeed, personal field observations in Spain, France, Austria, Croatia, and Iran suggest that hybrids/introgressed individuals are absent from well‐preserved floodplain forests (i.e., natural architecture and dynamic flooding).

For this purpose, we chose the Donau‐Auen National Park (DANP) as a model site. Among the five other major rivers in Europe which still host wild grapevine populations, none benefits from such an extensive protected forest area (93 km²) and an active dynamic flood pulse (Schnitzler & Carbiener, 2007). This Danube area also regroups all these parameters and contains the largest population of wild grapevine recorded in Europe (Arnold, 2002).

Since 1993, many studies have been conducted in the park on river dynamics, vegetation and target species like *Vitis*. In 2003, under the supervision of Christian Fraissl from the DANP, a comprehensive field survey was conducted in the entire protected area by Claudia Freiding, Christa Gußmark, and Ulrike Haubenwallner. From this study, we now know that there are exactly 180 grapevines in the DANP. Among them, non‐native *Vitis* were recorded.

Our study aimed to provide insight into the ecology of the *Vitis* complex in this Danube region through molecular analyses of cpDNA and nSSR regions, pedigree of grapevines, morphology, and distribution of *Vitis* individuals.

## 2 MATERIALS AND METHODS

### 2.1 Plant material

The Eurasian wild *V. vinifera* ssp. *sylvestris* is dioecious, with either male flowers with fully developed anthers and fertile pollen and a nonfunctional ovary, or female flowers with a large, well‐developed ovary and pistil associated with small anthers with sterile pollen. In male flowers, the pollen is heavy and sticky, which suggests that these plants are mainly pollinated by insects, or wind at short distances. Rare cases of hermaphroditism have nevertheless been reported (Anzani et al., 1990; Levadoux, 1956). In contrast to the wild grapevine, the domesticated form of *V. vinifera* is hermaphroditic and self‐compatible. Based on this reproductive difference, the two taxa were separated into two subspecies: *V. vinifera* ssp. *sylvestris* (Gmelin) Hegi and its domesticated relative *V. vinifera* ssp. *vinifera*.

The latter have been propagated vegetatively for centuries (Mylesa et al., 2011), leading to somatic mutations that have actively contributed to the increase in the number of grape varieties. Interspecific cultivars as well as PIWI are also hermaphrodites. Rootstocks on their side are mainly dioecious.

The *Vitis* taxa all over the world are light‐demanding large tendrillar lianas. They live and reproduce in gaps, upper canopies, bushes along erosive river banks, and the edges of temperate (alluvial) forests in the Northern Hemisphere. American *Vitis* have a naturally larger range of habitats than the unique Eurasian *Vitis*, with individuals situated at the extremes of environmental gradients for moisture and texture (Morano & Walker, 1995).

### 2.2 The study area

**Figure 2:**
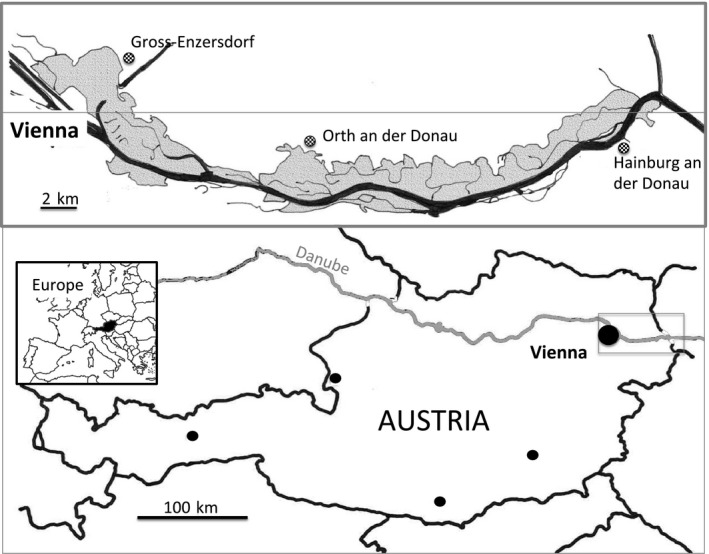
Localization of the study area Donau‐Auen National Park

The study area (48°8′0″N 16°55′0″E) covered 93 km² in lower Austria (Figure 2). The climate is temperate continental, with a mean temperature of 10°C and a mean annual rainfall of 600 mm. The Danube in Austria (350 km long) has kept an alpine hydrologic regime with the highest water levels between May and June. In addition, short episodic fluctuations throughout the year can occur. Since the 1870s, the flooded area has been reduced to a 3–7 km wide area within two dykes, leading to significant incision of the river within its floodplain. Floods have also become less erosive and less frequent, but fluctuations in the water levels are still high (7–9 m) within the dykes (Liepolt, 1965). Soils are calcareous, fine to coarse‐textured fluvisols.

Along the most dynamic parts of the river network, the floodplain forests are composed of mosaics of white willow (*Salix alba* L.), black poplars (*Populus nigra* L.) and white poplars *(Populus alba* L.). On the elevated terraces, the canopy is dominated by light‐demanding hardwood species such as oak (*Quercus robur* L.), ash (*Fraxinus excelsior* L.), white poplar and elm (*Ulmus minor* Mill.), and canopy liana (*Hedera helix* L., *Clematis vitalba* L., *V. vinifera* ssp. *sylvestris)*. These forests have traditionally been fragmented by numerous pathways for hunting (a total of 420 km long) and also include some permanent meadows.

Before becoming a single national park, the area included several types of protected areas. In 1978, the Lobau was designated as a protected area (Naturschutzgebiet). The Untere Lobau was included in a UNESCO Biosphere Reserve the same year. In 1979, the area called Donau–March–Thaya Auen received the status of Naturschutzgebiet. The area including Donau–March Auen and Untere Lobau became a Ramsar site in 1983. In 1996, the Donau‐Auen National Park (DANP) was created. This area was designated an IUCN category II National Park in 1997, and some of its areas are included in the Natura 2000 network. With the creation of the DANP, commercial forest management was abandoned, but the former forest management is still visible in the landscape, with variations according to the owners' practices. For example, Obere, Untere Lobau, and Mannswörth were administrated by Vienna, and the rest by the federal forest company. Globally, hybrid plantations were more frequent within the dykes, while oak plantations were more frequent outside the dykes. With regard to human practices in the more distant past, the DANP was managed in different ways, with regard to both river management and forestry. In the Unterer Lobau near Vienna, the flooding periods are long and frequent, with traditional extensive forest management. In the eastern part of the DANP, from Mannsdorf Under Donau to the Slovakian border, forests were intensively managed until the creation of the National Park.

### 2.3 Plant material sampling

One hundred and sixty‐five *Vitis* individuals (i.e., physically separated above ground) were found in the study area. Fifteen individuals could not be found or were not reachable. Each sample location was recorded by GPS. For each sample, we collected the following data: geographic coordinates, morphological data (number of stems at the base, DBH, and height of the main stem), and ecological data: number of host trees identified by species used for ascending, situation related to the dykes (within or outside) and habitat (forest edge with meadow, forest edge with channel or forest interior in a gap).

### 2.4 DNA extraction and amplification

The leaves collected from the 165 individuals were dried in silica gel. To identify hybrids, we added 21 cultivars and 19 rootstocks as an outgroup, all commonly cultivated in Austria and Europe. These included the hybrid Mgt 41b, which is a hybrid between a *V. vinifera* cultivar and *V. berlandieri*. The 19 rootstocks were from collections of the Institut für Rebenzüchtung Geilweilerhof (Germany) and from the Agroscope Viticulture Research Centre Pully (Switzerland).

Genomic DNA was extracted with the DNeasy Plant Mini Kit (Qiagen), according to the manufacturer's instructions. Twenty‐four microsatellites and five chloroplastic regions were amplified by PCR. Amplifications were carried out in 10 μl reactions containing 1x GoTaq Reaction Buffer, 0.75 mM MgCl_2_, 5 μg BSA, 0.25 mM dNTPs, 0.25 μM of each primer, 0.5 U GoTaqG2 DNA Polymerase (Promega), and 2–5 ng of template DNA. The PCR cycling conditions consisted of an initial activation step of 4 min at 94°C, followed by 30 cycles each of 60 s at 92°C, 50 s at 52–56°C (Appendix 1), and 60 s at 72°C, with a final extension step of 10 min at 72°C. Macrogen did the genotyping. Amplified fragment lengths were assigned to allele sizes with GeneMapper software v 3.7 (Applied Biosystems). Among the 24 pairs of markers, four markers (VVMD‐28, VMC‐5A1, VMC‐1C10, VVS2) did not amplify correctly. Five samples that did not amplify at least 15 pairs of markers were also removed from statistical analysis. All grape varieties and rootstocks amplified correctly. As a result, we retained 200 samples (i.e., 160 grapevines found in the wild; 21 cultivars; 19 rootstocks), analyzed with 20 microsatellite (nSSR) loci and five chloroplastic (cp) DNA loci.

### 2.5 Genotypes

We carried out a STRUCTURE 2.3.4 analysis on the 200 individuals (Pritchard, Stephens, & Donnelly, 2000). The following options were used: 10,000 burn‐in, 20,000 MCMC, admixture model and correlated allele frequencies. This method is based on the use of Markov Chain Monte Carlo (MCMC) simulations to infer the assignment of genotypes to *K* distinct clusters. The underlying algorithms attempt to minimize deviations from Hardy–Weinberg and linkage disequilibria within each cluster. In accordance with Evanno, Regnaut, and Goudet (2005), we did 10 iterations for each *K* value (*K *=* *1 to *K *=* *6). The most likely number of clusters (*K*) was estimated in Structure Harvester, using the maximum value of L(*K*) and calculating delta Δ*K*.

Private alleles are alleles that are found only in a single population among a broader collection of populations. They were calculated using the frequency‐based statistics of GenAlEx 6.5. (Peakall & Smouse, 2006). We checked for private alleles within the pure wild grapevines, cultivars and rootstocks. We also used these results for the identification of hybrid/introgressed origins.

### 2.6 Haplotypes

The cp DNA markers were used to determine: (1) the genetic characterization of hybrids and introgressed individuals, (2) the direction of hybridization, given that the cpDNA is inherited from the mother (Arroyo‐Garcia et al., 2006; Arroyo‐García et al., 2002), (3) the place where hybridization/introgression occurred (i.e., within or outside the DANP with, in the latter case, birds transporting seeds from the fields to the forest), and (4) the potential diversity of haplotypes in the wild grapevine population.

### 2.7 Genetic diversity and geographic structure (on the 20 SSR)

The distribution of the population tended to be aggregated at two levels. At the first level, a western group (Untere Lobau and Obere Lobau) was separated from an eastern group by 4 km, with the latter extending up to the Slovakian border. At the second level, there were five groups (Mannswörth, Eckartsau, Orth, Untere Lobau, Obere Lobau). To examine this geographic pattern, we used the individual‐based Bayesian clustering methods implemented in STRUCTURE 2.3.4. We investigated intraspecific population structure and admixture. We used an admixture model with allele frequencies correlated according to Evanno et al. (2005). Ten independent analyses were carried out for each number of clusters *K* (1 ≤ *K *≤* *26), with 80,000 MCMC iterations after a burn‐in of 20,000 steps.

#### 2.7.1 Focus on the true wild grapevine

To investigate the potential geographic structure, we performed complementary analysis with R ADEGENET package (Jombart, 2015; R Core Team 2013). The genetic diversity was assessed with GenAlEx 6.5 (Na, Ne, Ho, He). Clones were detected using GenAlEx and were confirmed in the raw data set.

#### 2.7.2 Focus on the hybrids

First, we identified the clones in the hybrid population and calculated the respective distances between them. Second, we removed them in order to analyze the full and half sibship assignments, as well as parent assignments of the hybrids/introgressed individuals. The analysis was performed in Colony 2.0 (2008; updated 2014 http://www.zsl.org/science/software/colony). We considered the hybrids as offspring, and the true wild grapevines, cultivars and rootstocks as putative parents. The following parameters were used: Mating system I: female polygamy/male polygamy, Mating system II: without in‐breeding/without clones; Species: dioecious/ diploid, Length of run: medium; Analysis method: full likelihood (FL), and Sibship size scaling: no prior. For the other parameters, we used the default values.

### 2.8 Morphology versus habitats

To investigate the influence of the flooding process on *Vitis* morphology, we compared statistically variations in DBH, height and number of stems between individuals within and outside dykes. As the data were not normally distributed, nonparametric tests (Mann–Whitney) were used. The same tests were used for investigating the relationships between ecological characteristics (flooding, habitat) on *Vitis* morphology.

## 3 RESULTS

### 3.1 Picture of the *Vitis* complex

**Figure 3:**
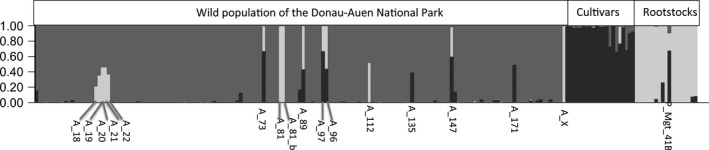
Population structure of the *Vitis* complex of the Donau‐Auen National Park inferred with the Bayesian clustering algorithm implemented in STRUCTURE. Each individual is represented by a vertical bar, partitioned into *K* segments representing the proportions of ancestry of its genome in *K* = 3 clusters

**Figure 4:**
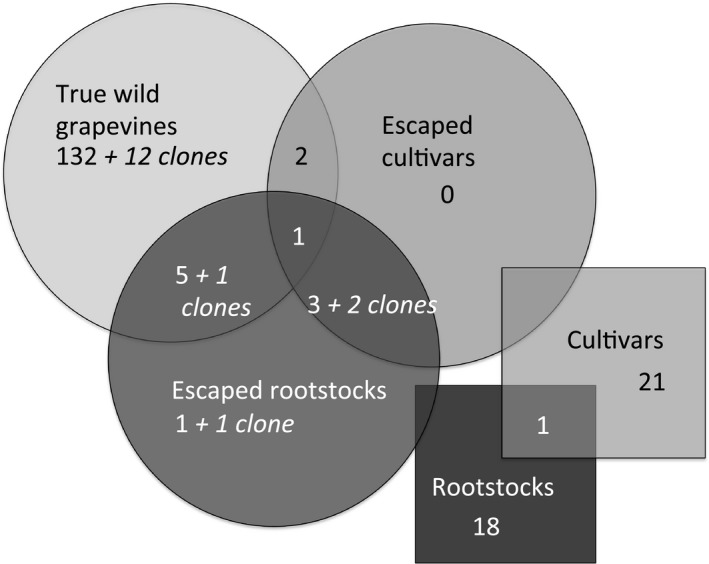
Distribution of the 160 studied individuals of the Donau‐Auen National Park (DANP) within the categories of True wild grapevines, escaped cultivars and escaped rootstocks. The circles contain the numbers of individuals and clones in the three categories. The squares contain the numbers of cultivars/varieties and rootstocks added to the study

The structure analysis (Figure 3) performed on the 200 grape samples suggested that two groups could be retained among the 160 individuals collected in the wild: one containing all *V. vinifera* subspecies and the other regrouping hybrid rootstocks. However, we retained *K *=* *3, separating the true wild grapevines (ssp. *sylvestris*) from cultivars (ssp. *vinifera*) and hybrid rootstocks. In the rootstock clade (in green), 41 B Millardet et de Grasset (41 B MGt) showed alleles of *V. vinifera*, which is normal as it was issued from a crossing between *V. vinifera* and *V. berlandieri*. In summary, of the 160 *Vitis* individuals collected in the wild from the DANP and analyzed, 144 *Vitis* were genetically different and 16 were clones. Among the 144 *Vitis* individuals, 132 were true wild grapevines and 12 were hybrids/introgressed individuals. Among the 12 hybrids, we found the following taxa: one rootstock × rootstock, five true wild grapevine × rootstock, three cultivar × rootstock, and one true wild grapevine × cultivar × rootstock. Clones were found in true wild grapevines (12) and crossings of rootstock × rootstock (1), true wild grapevine × rootstock (1), and cultivar × rootstock (2) (Figure 4).

#### 3.1.1 Haplotypes

We identified a total of five haplotypes distributed in both wild grapevines and hybrids (see 2.4 and 3.1). H1, which is common in the wild populations of western Europe; H2, which is common in the wild populations of eastern Europe; H3, which is similar to Chardonnay and Merlot; H4, which is similar to Chasselas and Cabernet Sauvignon as well as some rare true wild grapevines; and H5, which regrouped all the American rootstocks of various origins.

#### 3.1.2 Host trees

*Vitis* climbed on a total of 330 trees or shrubs belonging to 24 species in the DANP, the most frequently being *Cornus sanguinea* L. (20%), *Populus alba* L. (15%), and *Acer campestre* L. (14.7%). A single *Vitis* individual may use one to five different hosts.

### 3.2 Focus on the true wild grapevine

#### 3.2.1 Geographic structure

The analyses in STRUCTURE and ADEGENET revealed no geographic structure among the 132 true grapevines, despite the wide distribution of individuals. Concerning the global genetic diversity, all markers were polymorphic, with the number of alleles ranging from two to 12 according to the markers. The Shannon's Information Index was 0.8. The heterozygosity values ranged from 0.03 to 0.80. The mean heterozygosity value was 0.418, which was identical to the expected heterozygosity (0.418). (Table 1). Both results indicated a random mating population, with free gene exchanges.

**Table 1:** Summary of genetic diversity in the true wild grapevine population (144 individuals)

	Na	Ne	Ho	He	I
Mean	5.55	1.998	0.418	0.418	0.8
*SE*	0.555	0.213	0.047	0.046	0.089

Ho and He, observed and expected heterozygosities, respectively. I, Shannon's Information Index; Na, number of alleles; Ne, effective number of alleles.

#### 3.2.2 Clones

Twelve clones were found close to each other, within 2–36 m. Ten true wild grapevines produced clones. Eight of them were by pairs, and two of them by threes.

#### 3.2.3 Private alleles

Of 144 individuals (132 true wild and 12 clones) of the wild grapevine, we found 16 private alleles distributed on nine markers. In contrast, although the cultivars and rootstocks had reduced numbers of individuals, they had many more private alleles (respectively, 30 and 72) distributed on 15 and 18 markers of 20 (Table 2).

**Table 2:** Number of private alleles in the wild grapevines, cultivars, and rootstock

	Wild grapevines	Cultivars	Rootstocks
	*N* = 144	*N* = 21	*N* = 18
Nb alleles	16	30	72
Nb markers	9	15	18

*N*, total number of individuals.

#### 3.2.4 Haplotypes

The following haplotypes were found in the 132 individuals: H1, common in the wild populations of western Europe, was found in 128 individuals; H2, common in the wild populations of eastern Europe, was found in three individuals; and H4, commonly found in cultivars such as Chasselas and Cabernet Sauvignon, was also present in some of the true wild grapevines.

#### 3.2.5 Morphology versus habitat

**Figure 5:**
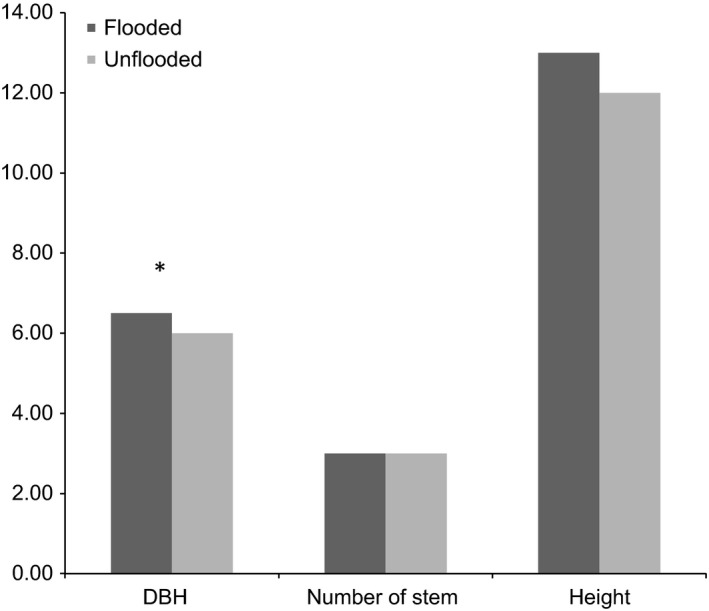
Comparison of ecological variables (DBH, total height, number of stems) in relation with the flooding process

Most individuals were found on the left side of the Danube in the study area. Eighty‐six individuals grew outside the dykes against sixty within dykes. Taking into consideration only the habitats, 42% of the true wild grapevines lived at the edges of forest/meadow, 33.3% at the edges forest/channels, and 23.9% in forest gaps. The Mann–Whitney test indicated that DBH varied significantly with the occurrence of flooding, with higher trunk diameters in flooding areas (Figure 5). The number of stems per individual depended on the habitat, with a higher number of stems in gaps and the edges of forests with channels (the mean number of stems was 4.8 and 5, respectively) than in the edges of forests with meadows (mean number of stems was 3). The total height of the grapevine was significantly higher in the gaps compared with the edges of forests with channels or meadows (*p* < .001).

### 3.3 Focus on hybrids/introgressed

#### 3.3.1 Orientation of crossings and parentage

Table 3 gives some of the characteristics of the hybrids. From the haplotypes, we were able to deduce the direction of hybridization. Eight had the H1 haplotype typical of the majority of *sylvestris* in western Europe; two had the H3 haplotype similar to Chardonnay and Merlot; two had the H4 haplotype similar to Chasselas, Cabernet Sauvignon, and some rare true wild grapevines; and four had the H5 haplotype regrouping all American rootstocks of various origins. We expected to find the parental origin from the genotypes. We found five types of hybrids.

**Table 3:** List of hybrids/introgressed individuals (haplotype, parentage, sex, clones, number of private alleles from wild grapevine, cultivar, or rootstock)

Individual number	Haplotype	Parentage	Sex	Clones	Number of private alleles from *V. sylvestris*	Number of private alleles from cultivars	Number of private alleles from roostocks
A_018	H1	True wild grapevine × rootstock	Presence of grapes		2		2
A_019	H1	True wild grapevine × rootstock	Unknown		1		3
A_020	H1	True wild grapevine × rootstock	Male	20 = 21			5
A_022	H1	True wild grapevine × rootstock	Unknown		1		4
A_112	H1	True wild grapevine × rootstock	Presence of grapes		2		7
A_135	H1	True wild grapevine × (Gruener Weltiner) vinifera	Presence of grapes		1	1	
A_171	H1	True wild grapevine × (Blaufrankisch) vinifera	Unknown		1	2	
A_089	H3	Vinifera × rootstock	Presence of grapes	89 = 97		2	6
A_096	H4	Vinifera × rootstock	Presence of grapes	96 = 73_b		2	4
A_X	H5	Riparia gloire (rootstock) × rootstock × vinifera	Unknown			1	13
A_081_b	H5	Rootstock × rootstock	Unknown	81_b = 81			16
A_147	H5	Rootstock × vinifera × true wild grapevine	Presence of grapes		1	1	7

##### True wild grapevine × rootstock

Among the six hybrids, five of them (18, 19, 20, 21, 22) were closely related (half‐sibs) and distributed along 300 m of an active channel of the Danube. They shared one allele per locus. According to the results of Colony, the putative mother may have been 6 km downstream on the edge of the forest and a channel. The confidence index was nevertheless too low. No. 112 was also a crossing between an unknown mother true wild grapevine and the pollen of rootstock.

##### True wild grapevine × cultivar (*vinifera*)

Two individuals were issued from a crossing of a true wild grapevine and a cultivar.

No. 135 was a crossing between female 99 and the Grüner Weltiner cultivar. No. 135 was located along a road within the national park, and the mother was located 100 m downstream along a dead arm. No. 171 was a crossing between female 144 and a Blaufrankisch cultivar. The mother was located 600 m upstream along a dead arm.

##### Cultivar × rootstock

Three hybrids were crossings between ssp. *vinifera* as the mother and a rootstock. No. 96 was at the edge of the DANP along a cultivated area: it has Cinsaut as a mother, and an unknown rootstock pollen. No. 89 and No. 97 (a clone of 89) were crossings including Baco Noir and Riparia Gloire. These two plants were also situated at the edge of the DANP.

##### Rootstock × rootstock

Three rootstocks were issued from various American taxa. No. 81 and No. 81b (a clone of 81) included *Vitis riparia* in the parentage. X had *Riparia* in both parents. All were situated along the main stream.

##### Rootstock × cultivar × wild grapevine

No. 147 had *V. riparia* parents but also Tinturina (identical to Usellina) in the genotype. It contained private alleles of wild grapevines. It was along the main stream in an industrial area.

#### 3.3.2 Clones

As mentioned, we found four clones among the hybrids/introgressed (Figure 4). All types of hybrids/introgressed forms were thus able to reproduce vegetatively. The maximum distance between two clones was about 350 m.

#### 3.3.3 Ecology

Hybrids were found within and outside the dykes, mostly on forest edges.

More precisely, most hybrids that included the genome of the true wild grapevine (18, 19, 20 and 21) were found close to each other along a branch of the main channel of the Danube. This part is active with erosive activity from flooding. The hybrids were present on a terrace along a sandy road. They had many stems, with up to 15 stems for No. 20. They all presented dense foliage from the ground up to 20 m. No. 22 was located close to Orth an der Donau along a pathway commonly used by bikers, and not far from the vineyards at the border of the park. No. 96, 112, 135 and 171 were located along a road close to an ancient main branch of the Danube, which was still connected to the main stream when widespread flooding occurred. The hybrids including those issued from crossings between rootstocks (X, 81, 81_b, and 147) were mainly established along the main channel of the Danube. Given the low number of hybrids, no statistical analysis was performed. Hybrids/introgressed individuals were vigorous with a deep cover of foliage up to 20 m high. They had many stems at the basis, and two of them were young individuals. Ten of 16 produced flowers and fruits. They were dioecious or hermaphrodite. In spite of their vigor, these non‐native taxa of grapevines had penetrated into forest gaps or massively invaded the anthropized sites of the DANP.

## 4 DISCUSSION

Our study pointed out a variety of *Vitis* taxa including endangered native species and hybrids with cultivars and escaped rootstocks. The number of true wild grapevines can be interpreted as the consequence of relatively suitable ecological conditions (e.g., maintenance of flooding events, large forest cover) compared with other populations of Europe (Spain, Portugal, Italy, France), which are now reduced to a few individuals with a significant reduction in the observed heterozygosity (Andrés et al., 2012; Di Vecchi‐Staraz et al., 2008; Grassi et al., 2003; Lopes, Mendonça, Rodrigues dos Santos, Eiras‐Dias, & da Cămara Machado, 2009).

The relatively high genetic diversity of true wild grapevines seems to be a legacy from the beginning of the Holocene when Danubian populations received genes from two migrating populations, one originating in southern Italy and moving northward via the Alps into central Europe, the second originating in the Balkan area and migrating westward (Grassi, De Mattia, Zecca, Sala, & Labra, 2008; Taberlet, Fumagalli, Wust‐Saucy, & Cosson, 1998). This legacy was kept in the genome of the population for millennia, until the 19th century.

The heterozygosity was lower than that observed in other *Vitis* populations of Europe (Arnold, Schnitzler, Parisot, & Maurin, 2009; Bodor et al., 2010; Zoghlami et al., 2013), but the genetic diversity was still quite high. Of course, the current situation is far from optimal if we consider the historical reports (see 1). The low survival can easily be explained by the conditions generated by embankment, which has destroyed suitable sites for the establishment of young plants, such as upper‐forested terraces. A second factor that may explain both the low densities and perhaps the clustering of the current population is the past forest management, which became more intensive after river regulation, with forest managers removing the climbers. A third factor is the low regeneration potential. According to observations by the DANP staff, seedlings may be abundant in spring, but they disappear quickly over the year. The sinking of the water table has induced dryness in the top layers of the soil, making it unsuitable for the survival and development of young plants. Another consequence of the sinking water table is that the typical fluvisols currently found in the area have already started to evolve (Arnold, 2002), similar to the observed shifts in plant communities. Along rivers with altered disturbance regimes, tree communities no longer belong to the same plant community as their understorey (Roulier, 1998; Roulier, Teuscher, & Weber, 1999) and seedlings of grapevines are not part of these plant communities.

The range of lengths and diameters was found rather high among adult grapevines. The larger diameters found in forest gaps and edges between forests and channels within the dykes can be explained by good conditions of light, nutrient, and moisture. These individuals invested their efforts in a single stem in order to reach the canopy rapidly, in particular when gaps are small and surrounded by tall trees. Single stems are also the result of growth without any trauma such as breakage following host fall.

The gene pool of the naturalized grapevines found in the DANP shows high genetic diversity due to genetic admixture among different taxa. The detailed pedigree reconstruction of the hybrids/introgressed *Vitis* allowed us to prove that the hybridization pattern is thus symmetric in nature. In viticulture, artificial bidirectional interspecific crossing has been successful and the development of these crossings is ensured by human care. Yet it has never been demonstrated that this could spontaneously occur. Our study also showed that hybrids involving rootstock genes were established preferentially along ancient main branches of the Danube, which are sometimes quite active, or along the main stream. Another interesting result is that hybrids and introgressed individuals were not so abundant in this area and did not succeed in penetrating the forest interior. Perhaps the competitivity of native plant species in the understorey, shown through architectural and phytosociological studies (Schnitzler, 1994), has prevented their establishment or, like the native grapevines, they cannot integrate the changing plant environment. There are certainly additional causes, such as strict governance regarding the cleaning of vineyard peripheries. This would reduce the feral propagule pressure.

Based on our results and the literature, we can conclude that the current population of wild grapevine of the DANP is one of the last bastions of the former vast metapopulation that extended throughout Europe. This area has maintained enough suitable habitats to preserve true wild grapevines from attacks by American pests and diseases, thanks to the accessibility of groundwater to roots and the maintenance of flooding, the preservation of a large forest cover, and the strict protection of the species. These results are of great importance for conservation biology. However, as dynamic floods seem to have gone forever from large river plains, the establishment of native offspring is probably impossible. If a re‐wilding strategy is considered in the DANP (i.e., re‐creation of erosive zones along the main river and adjacent channels), one should take into account that hybrids may take advantage to this new situation. This is, however, the only chance for wild grapevine populations to regenerate. Whatever the case, re‐wilding actions must not only address protection of one specific subspecies, even endangered, but they must also consider the interest of the global ecosystem functioning. We thus hope for the return of erosive floods in a not too distant future.

## DATA ACCESSIBILITY

Material can be obtained at the DANP in Orth and der Donau. Other data are archived at the University of Lausanne.

## AUTHOR CONTRIBUTIONS

Dr. Claire Arnold directed the study, did part of the fieldwork, and wrote part of the article. Mag. Olivier Bachmann collected the samples in the collections and the Donau‐Auen National Park, and he did the laboratory analysis and the statistics. Prof. Dr. Annik Schnitzler, went to the field, did the ecological part of the study, and wrote part of the article.

## APPENDIX 1

### 1.1

**Table A1:** List of 24 nSSR and 5 cpDNA primers, references, and annealing temperatures. (In gray and italic primers that did not amplify correctly)

Primer	Reference	Cycles
VVMD 5	Bowers, Dangl, Vignani, & Meredith, 1996	94°C‐4 min; 30 cycles (92°C‐60 sec, 54°C‐50 sec, 72°C‐60 sec) 72°C‐10 min
VVMD 7	Bowers et al., 1996	94°C‐4 min; 30 cycles (92°C‐60 sec, 52°C‐50 sec, 72°C‐60 sec) 72°C‐10 min
VVMD 8	Bowers et al., 1996	94°C‐4 min; 30 cycles (92°C‐60 sec, 56°C‐50 sec, 72°C‐60 sec) 72°C‐10 min
VVMD 17	Bowers, Dangl, & Meredith, 1999	94°C‐4 min; 30 cycles (92°C‐60 sec, 56°C‐50 sec, 72°C‐60 sec) 72°C‐10 min
VVMD 24	Bowers et al., 1999	94°C‐4 min; 30 cycles (92°C‐60 sec, 56°C‐50 sec, 72°C‐60 sec) 72°C‐10 min
VVMD 25	Bowers et al., 1999	94°C‐4 min; 30 cycles (92°C‐60 sec, 53°C‐50 sec, 72°C‐60 sec) 72°C‐10 min
VVMD 26	Bowers et al., 1999	94°C‐4 min; 30 cycles (92°C‐60 sec, 56°C‐50 sec, 72°C‐60 sec) 72°C‐10 min
VVMD 27	Bowers et al., 1999	94°C‐4 min; 30 cycles (92°C‐60 sec, 56°C‐50 sec, 72°C‐60 sec) 72°C‐10 min
*VVMD 28*	*Bowers* et al.*,* 1999	*94°C‐4 min; 30 cycles (92°C‐60 sec, 56°C‐50 sec, 72°C‐60 sec) 72°C‐10 min*
VVMD 31	Bowers et al., 1999	94°C‐4 min; 30 cycles (92°C‐60 sec, 53°C‐50 sec, 72°C‐60 sec) 72°C‐10 min
VVMD 32	Bowers et al., 1999	94°C‐4 min; 30 cycles (92°C‐60 sec, 56°C‐50 sec, 72°C‐60 sec) 72°C‐10 min
VVMD 36	Bowers et al., 1999	94°C‐4 min; 30 cycles (92°C‐60 sec, 56°C‐50 sec, 72°C‐60 sec) 72°C‐10 min
VrZAG 62	Sefc et al. 1999	94°C‐4 min; 30 cycles (92°C‐60 sec, 56°C‐50 sec, 72°C‐60 sec) 72°C‐10 min
VrZAG 62	Sefc et al. 1999	94°C‐4 min; 30 cycles (92°C‐60 sec, 56°C‐50 sec, 72°C‐60 sec) 72°C‐10 min
*VMC 1C10*	*Vitis Microsatellite Consortium*	*94°C‐4 min; 30 cycles (92°C‐60 sec, 56°C‐50 sec, 72°C‐60 sec) 72°C‐10 min*
VMC 2A5	*Vitis* Microsatellite Consortium	94°C‐4 min; 30 cycles (92°C‐60 sec, 56°C‐50 sec, 72°C‐60 sec) 72°C‐10 min
VMC 2B3	*Vitis* Microsatellite Consortium	94°C‐4 min; 30 cycles (92°C‐60 sec, 56°C‐50 sec, 72°C‐60 sec) 72°C‐10 min
VMC 2C3	*Vitis* Microsatellite Consortium	94°C‐4 min; 30 cycles (92°C‐60 sec, 56°C‐50 sec, 72°C‐60 sec) 72°C‐10 min
VMC 2H4	*Vitis* Microsatellite Consortium	94°C‐4 min; 30 cycles (92°C‐60 sec, 56°C‐50 sec, 72°C‐60 sec) 72°C‐10 min
VMC 4G6	*Vitis* Microsatellite Consortium	94°C‐4 min; 30 cycles (92°C‐60 sec, 56°C‐50 sec, 72°C‐60 sec) 72°C‐10 min
*VMC 5A1*	*Vitis Microsatellite Consortium*	*94°C‐4 min; 30 cycles (92°C‐60 sec, 56°C‐50 sec, 72°C‐60 sec) 72°C‐10 min*
VMC 5C5	*Vitis* Microsatellite Consortium	94°C‐4 min; 30 cycles (92°C‐60 sec, 56°C‐50 sec, 72°C‐60 sec) 72°C‐10 min
VMC 5H2	*Vitis* Microsatellite Consortium	94°C‐4 min; 30 cycles (92°C‐60 sec, 56°C‐50 sec, 72°C‐60 sec) 72°C‐10 min
*VVS 2*	*Thomas & Scott* 1993	*94°C‐4 min; 30 cycles (92°C‐60 sec, 54°C‐50 sec, 72°C‐60 sec) 72°C‐10 min*
VndhF1	Bachmann & Arnold in prep	94°C‐4 min; 30 cycles (92°C‐20 sec, 51°C‐20 sec, 72°C‐60 sec) 72°C‐10 min
VndhF2	Bachmann & Arnold in prep	94°C‐4 min; 30 cycles (92°C‐20 sec, 51°C‐20 sec, 72°C‐60 sec) 72°C‐10 min
VtrnK‐1	Bachmann & Arnold in prep	94°C‐4 min; 30 cycles (92°C‐20 sec, 53°C‐20 sec, 72°C‐60 sec) 72°C‐10 min
VtrnK‐2	Bachmann & Arnold in prep	94°C‐4 min; 30 cycles (92°C‐20 sec, 49°C‐20 sec, 72°C‐60 sec) 72°C‐10 min
VtrnC	Bachmann & Arnold in prep	94°C‐4 min; 35 cycles (92°C‐20 sec, 51°C‐20 sec, 72°C‐60 sec) 72°C‐10 min
